# Antibiotic use by backyard food animal producers in Ecuador: a qualitative study

**DOI:** 10.1186/s12889-022-13073-4

**Published:** 2022-04-08

**Authors:** William F. Waters, Martin Baca, Jay P. Graham, Zachary Butzin-Dozier, Lenin Vinueza

**Affiliations:** 1 Universidad San Francisco de Quito, Diego de Robles S/N y Pampite, Cumbayá, Quito Ecuador; 2 School of Public Health, University of California, Berkeley, CA USA

**Keywords:** Antibiotics, Ecuador, Food animal production, Qualitative research

## Abstract

**Background:**

Antibiotics are increasingly used throughout the world in food animal production for controlling and preventing disease and for promoting growth. But this trend also has the potential for promoting antibiotic resistance, which represents a threat to human, animal, and environmental health. The use of antibiotics and the potential effects of antibiotic dependence has often been associated with large-scale food animal production. But rural households also engage in small-scale production, often operating literally in backyards. While some small-scale producers use veterinary antibiotics, many do not. This paper examines knowledge, attitudes, beliefs, and agricultural practices (KAP) that represent an alternative to dependence on antibiotics.

**Methods:**

Qualitative field research was based on four focus group discussions (FGDs) with non-indigenous backyard food animal producers in four communities near Quito, Ecuador and two FGDs with veterinarians. FGDs were supplemented by structured observations and key informant interviews. They were recorded with digital audio devices and transcriptions were analyzed independently by two researchers using a three-stage coding procedure. Open coding identifies underlying concepts, while axial coding develops categories and properties, and selective coding integrates the information in order to identify the key dimensions of the collective qualitative data.

**Results:**

Backyard food animal producers in the Ecuadorian highlands generally do not use antibiotics while rearing small batches of animals and poultry for predominantly non-commercial household consumption. Instead, they rely on low cost traditional veterinary remedies. These practices are informed by their Andean history of agriculture and a belief system whereby physical activity is a holistic lifestyle through which people maintain their health by participating in the physical and spiritual environment.

**Conclusions:**

Backyard food animal producers in the Ecuadorian highlands implement complex strategies based on both economic calculations and sociocultural underpinnings that shape perceptions, attitudes, and practices. They use traditional veterinary remedies in lieu of antibiotics in most cases because limited production of food animals in small spaces contributes to a predictable household food supply, while at the same time conforming to traditional concepts of human and environmental health.

## Background

The use of antibiotics in food animal production for controlling and preventing disease and for promoting growth is common throughout the world. An estimated 63,151 tons of antibiotics were used in food animal production worldwide in 2010, and use is expected to increase by 67 percent to 105,596 tons by 2030 [1]. This trend has been termed “ominous” [2] because of the potential for promoting antibiotic resistance, which represents a threat to human, animal, and environmental health worldwide. While the use of antibiotics may enhance productivity, it also creates the potential for spreading multidrug-resistant bacteria throughout the food chain [3] and in settings where domestic animals live in close contact with workers, members of nearby households, and inhabitants of surrounding communities [4–6]. The intensification and concentration of food production; the globalization of marketing, transportation, and commerce; and evolving patterns of worldwide consumer demand have tended to accentuate the use of antibiotics. Consequently, antibiotic resistance is associated with 700,000 annual deaths globally, with a projected loss of 10 million lives by 2050 [7].

The dependence on antibiotics and the potential for antibiotic resistance in commercially oriented large-scale food animal production has received substantial attention [8, 9]. But at least 450 million rural households engage in small-scale agricultural production worldwide, constituting 85 percent of farms worldwide [10]. The farming systems of these low-resource, small-scale agricultural producers often include poultry and other food animal species as a critical component of household food security [11]. These systems also often incorporate complex mixes of productive and income-generating activities based on wage labor, petty commerce, and transportation [12]. While backyard producers may occasionally engage in sale, gifting, or exchange, they keep animals—literally in their back yards—principally for household consumption, using the least amount of financial and material resources necessary.

This strategy functions largely beyond commercial circuits and is not usually designed to generate profit. Little or nothing is invested in infrastructure, equipment, or materials [13] and the purchase of equipment and materials is minimal or entirely absent; poultry are kept at night in makeshift coops constructed of leftover or recycled building materials such as plywood and corrugated metal roofing; during the day, chickens and ducks forage in the backyard for insects, seeds, household scraps, and the like. In the Andean region, many backyard producers also keep guinea pigs in rustic hutches. They are fed with alfalfa and used for sale or home consumption and have cultural value, principally as gifts. One or two sheep, goats, or pigs may be staked out on roadsides or grazed in nearby lots. Cattle are rarely kept by backyard producers because they require access to larger amounts of land. In sum, backyard production is part of a household subsistence strategy that also includes cash income earned by some family members as well as limited production of vegetables, tubers, and grains in what amounts to large gardens.

The use of antibiotics by small-scale food animal producers has been noted in low- and middle-income countries [14, 15] and is often influenced by information and misinformation provided by veterinary product vendors [16, 17]. But when contact with veterinarians is limited for reasons discussed below, many small-scale producers depend on advice from family members and neighbors to inform their decision-making, and they use traditional veterinary remedies made from local plants and other readily available ingredients that are often found in the kitchen [18, 19]. Hence, while overall global trends suggest that food animal production increasingly intensive and based on maximizing productivity through the use of antibiotics, alternative practices that do not depend on antibiotics are also observable.

Throughout the Andean region of South America, small-scale, basically subsistence-oriented agriculture and food animal production represent important parts of the rural landscape, while adapting to emerging conditions and opportunities [20]. In the Ecuadorian highlands, small-scale, limited resource food animal production persists even though large-scale agribusiness has penetrated the region [21], and some small-scale producers engage in intensive, commercially oriented production [22, 23] and also in the face of transnational migration from rural areas [24]. But many rural households in Ecuador engage in small-scale food animal production using limited resources and inputs and may base their decisions and practices on traditional concepts of environmental and human health [25].

This paper reports on focus group discussions (FGDs) with backyard food animal production in four peri-urban communities in the Ecuadorian highlands near the capital city of Quito. These towns are formally considered rural parishes, and their residents have for decades continued to engage in small-scale backyard food animal production and agriculture, while at the same time maintaining close links to Quito for work, health care, and commerce [26]. In addition, FGD with veterinarians and key informant interviews (KI) were conducted. Recent studies conducted in these communities have documented the use of antibiotics in food animal production as well as the potential health and environmental impacts [6, 14]. These studies estimate that around 20 percent of small-scale food animal producers report using antibiotics [27, 28] with misuse being attributed to lack of information or knowledge [29] and to aggressive or even unscrupulous sales tactics by vendors of veterinary products [27].

The article addresses the question of why most backyard food animal producers do not use antibiotics. The aim is to understand the knowledge, attitudes and practices related to the prevention and control of diseases by backyard food animal producers in peri-urban towns near Quito, Ecuador as an alternative to dependence on veterinary antibiotics.

This paper complements existing research that shows that decision making among food animal producers is influenced by personal beliefs and perceptions [30, 31]. In some countries, the perceived benefits, availability, and low cost of antibiotics favor their use, and may be associated with risk aversion [32]. On the other hand, by studying the knowledge, attitudes, beliefs, and practices of backyard food animal producers in highland Ecuador, this study builds upon past research on producers who do not use antibiotics to raise their animals. Their practices may then represent alternatives for reducing inappropriate antibiotic use in this sector.

## Methodology

### Study design

The study was based on the grounded theory framework pioneered by Glaser and Strauss [33] and refined in later iterations [34]. Research based on grounded theory involves purposive sampling, data collection (including recording), and a multistage process of analysis that identifies categories of responses, determines key categories or dimensions, and develops a unified interpretation. Thus, beginning with subject selection conducted through theoretical sampling based on knowledge of or experience with the phenomena of interest, an iterative process of ongoing comparative analysis that provides the basis for the abstraction of basic concepts [35, 36]. Data collection continued until saturation is reached; that is, until no new information is obtained. In addition to saturation, a second principle of qualitative research is triangulation, by which findings are verified and validity is optimized by repeated applications of several complementary data collection techniques [33, 34]. The present study is based on data collection over a six-month period in 2019 using a cross-sectional research design and incorporates findings from: (i) four focus group discussions (FGDs) conducted in four communities with backyard food animal producers who varied in terms of sex, age, and level of education; two FGDs with veterinarians who work in food animal production; (iii) two key informant interviews with persons who were knowledgeable about backyard production; and (iv) structured observations of production facilities and processes.

The research consisted of six FGDs, two key informant interviews, and structured observations. The focus group is a planned and directed conversation designed to elicit perceptions, opinions, and insights from several people who share at one or more characteristics relevant to the study. The guided discussion provides a secure and confidential environment in which participants express their ideas in response to those of other participants. The purpose is not to arrive at consensus, but rather to understand the breadth and depth of opinions related to the research topic. Key informant (KI) interviews complemented focus group discussions by providing in-depth insights from individuals who are knowledgeable because of their personal or professional relationship to the topic of interest. A check list of structured observations provided additional information and were based on previous research in the study area [27, 28]. The observations included variety and number of species and the use of materials, equipment, and inputs.

A set of codes were developed prior to the initiation of field work based on previous research in the study area on the same topic [27, 28]. These codes were used to develop the question guides and then to form the basis for analysis in terms of five categories: (i) demographic and socioeconomic characteristics of producers including other sources of household income sources; (ii) characteristics of backyard food animal production (species and numbers); (iii) use of the animals (home consumption, sale, gifting); (iv) use of traditional home remedies; and (v) use of and sources of veterinary inputs (including antibiotics) and veterinary services. Verbatim transcriptions were prepared from audio recordings and analyzed using a systematic three-stage coding procedure. Transcriptions were manually and independently coded by two researchers followed by iterative meetings to arrive at consensus and final coding [34].

Open coding identified basic concepts expressed by study participants in their own terms: characteristics of backyard production (including species and numbers), reasons for keeping animals, use of antibiotics and other veterinary products as well as veterinarian services, and use of traditional inputs. Axial coding developed underlying categories and properties related to knowledge, attitudes, and practices of the dimensions identified by open coding. Selective coding integrated and refined the themes and the relationships among them and produced the principal dimensions discussed below: characteristics of operation, antibiotic use and alternatives, knowledge of antibiotic resistance and its consequences, and government policy. Table 1 summarizes the coding scheme.

**Table 1:** Coding scheme

Coding stage	Dimensions
Open coding	Characteristics of backyard production (including species and numbers)
	Reasons for keeping animals
	Use of antimicrobials and other veterinary products
	Use of veterinarian services
	Use of traditional inputs
Axial coding	Knowledge, attitudes, and practices
Selective coding	Characteristics of operation
	Antimicrobial use and alternatives
	Knowledge of antibiotic resistance and its consequences
	Government policy

### Setting

FGDs were conducted with backyard producers in the communities of Yaruqui, Otón de Velez, Checa, and Pifo, located approximately 40 km east of the capital city of Quito in the Ecuadorian highlands. Two FGD were conducted in Quito with veterinarians who work with livestock producers. A key informant interview (KI) was conducted with a specialist in livestock production and veterinary practices in the Ministry of Agriculture and Livestock in Quito. A second KI was conducted with a backyard producer in the community of Tumbaco, who was identified by the principal investigator. None of the FGD or KI participants identified themselves as indigenous.

### Respondents and recruitment

Backyard food animal producers were identified and recruited by an informal local leader in conjunction with the research team. She had no official position but was familiar with the families who live in the area based on participation in community activities. Meeting dates and times were agreed upon and confirmed, and the group discussions were held in the respective communities, each lasting approximately two hours. Participants in the veterinarian FGDs were recruited through professional networks.

### Ethics approval

FGD participants provided written informed consent, while the KI participants provided verbal informed consent. The study was approved by the IRBs of the Universidad San Francisco de Quito and the University of California, Berkeley.

## Results

The four FGDs conducted with backyard producers included a total of 41 participants recorded information on sex, age, education, and community of residence. Age ranged from 18 to 65 years of age; 85% were female, and the most common occupation was stay-at-home parent/spouse (46%); others work in commerce or services. 61% of the producers had less than a secondary education, which is about the national average for this age group.

The two veterinarian FGDs were conducted with a total of 18 participants and included information on sex, age, and education. 83% were male and all had undergraduate or graduate university training. These participants reported working in private practice, in government positions, or both. The two key informants were a female backyard food animal producer and a female technician in the veterinary division of the Ministry of Agriculture and Livestock.

The qualitative coding procedure described above identified four key dimensions related to knowledge, attitudes, and practices relevant to the use of antibiotics by backyard food animal producers: (i) characteristics of operation, (ii) use of antibiotics and alternatives, (iii) knowledge of antibiotic resistance and its consequences, and (iv) government policies.

### Characteristics of agricultural operation

Participants in the producer FGDs explained the characteristics of backyard food animal production and how they differ from commercially oriented small-, medium-, and large-scale production. Firstly, as discussed in greater detail below, they rarely use antibiotics or any other veterinary products. The majority reported that they never do. Secondly, according to FGD participants, and confirmed by KI participants and structured observation, backyard producers most commonly keep one of more of the following: between five and 20 chickens and/or guinea pigs and one or two sheep or pigs. Only four participants reported that they have cows because most do not have enough land to sustain them. Thirdly, they invest little in infrastructure such as coops or fences, instead opting for recycled or left-over materials such as boards and metal sheeting.

Producer FGD participants explained their management practices in terms of their motivation for engaging in backyard production. Their principal interest is not profit; backyard production is sold only sporadically if there is a special need for income in addition to what household members earn outside of the home or when the number of animals exceeds available resources-especially space. Rather, food animals are kept mainly for household consumption as an important complement to the household’s food purchased by a generally limited cash income, given that the minimum wage in Ecuador lies around USD 425 per month. For this reason, available resources are often used to feed backyard animals and poultry rather than relying exclusively on purchased animal feed.

Poultry production is almost universal because it requires very little investment or space and because meat and eggs are produced relatively quickly. Producer FGD participants also reported that they occasionally give chickens to family members, friends, or neighbors or may trade them for something of equivalent value. Larger species such as pigs are kept for longer periods and are often consumed during year-end celebrations. Participants in the veterinarian FGDs confirmed that for backyard producers, the major reason for keeping animals is household consumption. They also confirmed, as discussed below, that veterinarian services are rarely requested by backyard food animal producers.

FGD participants explained that in contrast to their backyard production, other small-scale producers have a more commercial orientation. While the cutoff point between backyard- and commercial small-scale operation is blurry, the major difference is that the latter invests more in infrastructure even though operations are not large, and in order to maximize production, they may seek the services of veterinarians and use veterinary products, including antibiotics. One veterinarian explained the difference in practice in this way, referring to his customers:

… it is customary for our people to have their animals in a feeding system of between five and 20 birds, as what we call backyard birds. Small producers are people who have small sheds and a fully developed infrastructure, but who are ultimately engaged in chicken production as a business or as a way of subsisting (male veterinarian, group 1, Quito).

These commercially oriented small-scale operators may consume part of what they produce, but that aspect is incidental to sales, which are the primary or only source of household income.

Participants in the veterinarian FGDs were more familiar with practices of medium- and large-scale food animal producers. Medium scale producers were described as having businesses that are large enough to provide sufficient household income without having to recur to other sources, but with modest investments in infrastructure and salaries. In contrast, large-scale producers maintain a larger number of animals (for example, more than 1,000 chickens), and substantial investment is dedicated to salaries, infrastructure, and veterinary products including antibiotics, which are used for disease prevention and control as well as growth promotion. One veterinarian explained the difference between these levels of production practices:

There are certain definitions or parameters that you must use to define the small-, medium- or large-scale producer of animals. A small-scale producer, I do not have the exact data, but a small producer is one who has a livelihood and something to live on. A medium-scale producer is one who has a business established and lives on his milk or meat production. A large-scale producer is one that already produces thousands of animals (male veterinarian, group 2, Quito).

In sum, backyard food animal producers are similar to small-scale subsistence agricultural producers, who are also found throughout the rural Ecuadorian highlands. In both cases, the essential characteristic of production is household subsistence, which necessarily must be supplemented by some source of cash income.

### Antibiotic use and alternatives

Few participants reported using antibiotics to prevent or cure disease or to promote growth, and none on a regular basis. On the few occasions when they are used, antibiotics are purchased in local commercial veterinary supply shops without prescriptions, often as a last resort when animas are sick and traditional methods do not have the desired effect [27]. For example, the producer KI is a single mother in her mid-40 s who works full time as a maid. After work, she tends to approximately 15 chickens, five ducks and ten rabbits in her backyard, where there is also a small garden where she grows vegetables for home consumption. The chickens and ducks provide eggs for home consumption and occasional sale or gifting; on rare occasions, she sells a chicken, duck, or rabbit. A few miles away, she works a small plot of corn with family members. This KI reported that she has never purchased or used veterinary products of any kind. The only chemical that she has used is household repellant to rid coops of fleas and other insects. She cannot afford other inputs, nor does she think they are necessary. With few exceptions, producer and veterinarian FGD participants concurred with this view.

Backyard food animal producer participants in the FGDs identified three principal factors related to the use of antibiotics in backyard production. Firstly, given their limited household cash incomes, the cost of these products is often prohibitive. From the perspective of scale of production and the reasons for engaging in backyard production, an empirical cost–benefit analysis suggests that buying antibiotics does not make sense because a single diseased animal can be slaughtered and consumed immediately and because there is no reason to promote accelerated growth. A veterinarian FGD participant explained that with regard to practice:

… one of the great advantages of backyard poultry production is that there is no pressure… to grow at 65 grams per day or have 14 to 15 birds per square meter. So, these are birds that do not have production stress and therefore they are animals that are not very challenged., so in short, the consumption of antibiotics at this level is very limited … (female veterinarian, Quito, group 3).

For that reason, veterinarian FGD participants explained that backyard producers rarely seek professional advice largely because of the cost and because these producers feel they do not need advice, as they prefer to base their practices on their own experience or advice from friends or neighbors. As one female producer in Oton de Velez explained, “the years of experience one has, one knows what illness it is and how to control it.” As a male producer in Pifo said, “my grandmother taught me that you should open the hen’s beak and put in a drop of lemon juice for the flu.”

Secondly, producers explained that antibiotics do not always work, so that practices involving traditional home remedies are used at little or no cost. According to a female participant in Checa:

For example, there are some antibiotics that when the animals have fever, they have no effect, so when a cow or bull has fever and the temperature doesn’t go down, we have to help it with liquified squash or cucumber because the fever doesn’t go down.

Thirdly, some producers explained that they abstain from using antibiotics on their animals because they believe them to be harmful for their animal’s health and, ultimately, for the health of the humans (including themselves) who consume those animals. As a female producer in Pifo explained this belief: “you can’t eat the meat because it is intoxicated, and it intoxicates you.” Producer FGD participants reported that in their general experience, meat that comes from animals or poultry that have been treated with antibiotics and other veterinary products have a medicinal flavor to the extent that to the extent that the meat cannot be consumed at all. They further explained their belief that an animal that has received too much medication is considered “poisoned.” Therefore, consuming it can produce illness in themselves and their families.

A female producer in Pifo explained that “sometimes an antibiotic is very strong and it damages the organs of the animals and persons.” This belief is related to consumption patterns and preferences of rural residents, who generally purchase and consume unprocessed foods in local shops and markets rather than processed and ultra-processed products sold in supermarkets in urban areas [37].

As an alternative to administering antibiotics, it is common-and nearly universal-that backyard producers use traditional home remedies for disease prevention and control. Producer FGD participants described practices based on using chili pepper, onion, garlic, or lemon juice mixed with water and applied in drops. As one female participant in Oton de Velez explained: “I put lemon in [the water] with chili and with that, the flu is gone.” These practices are shared among family members, friends, and neighbors, and only when traditional remedies do not achieve the expected results did participants report that they might use “modern” medicines, and in these cases, they base their practices on personal experience and recommendations from friends and family to choose-usually the least expensive option.

Sure, you buy the least expensive option because buying eighty or a hundred [doses] already costs more money, so you only buy twenty (female producer, Checa).

The use of traditional remedies by these backyard producers to treat illness in food animals conforms to a great degree to Andean cosmology, which is important even in communities like those discussed here that do not identify as indigenous [38]. According to perspectives elucidated in studies of ethnomedicine, reverence for Pachamama (literally “Mother Earth” in the Kichwa language), the physical and spiritual worlds are intimately intertwined, and human, animal, and environmental health are encountered and addressed in everyday life using traditional knowledge of medicinal plants and other materials found in nature [38]. Hence, small scale agricultural and livestock producers in these communities, as elsewhere in the Ecuadorian highlands, tend to eschew chemical inputs not only because of the economic costs, but because of beliefs related to the care of *Pachamama* [39, 40]. The use of ingredients found in the home, like the use of medicinal plants to treat human maladies, thus makes medical and ethical as well as economic sense. A male producer in Yaruqui explained that in practice:

When an animal has fever, I realize myself that it is preferable to bathe it or put it in the shade so it gets better, and I look for a home remedy for a fever because if I inject it, it will die.

By contrast, veterinary FGD participants reported that commercially oriented medium- and large-scale production practices are characterized by the indiscriminate use of antibiotics. As one explained:

People buy a box of a hundred chickens and ask for vitamins and antibiotics, and they are told that they do not need them, ... There are even people who buy vaccines and an antibiotic for the chicken flu as soon as it is sold. So, we usually tell [customers] that if their poultry doesn’t have the flu, why should they medicate? And they usually medicate after 28 days or in the fourth week. There are people, … if you don't sell it, they go to another store and buy (male veterinarian, group 2, Quito).

### Knowledge of antibiotic resistance and consequences

Producer FGD participants demonstrated that they possess a certain level of understanding about antibiotic resistance, which informs their decision to use those products or not. A female participant in Yaruqui explained her belief that “what happens is that just like humans, when the animals are incorrectly treated, the disease becomes resistant.” Some participants believed that excessive use leads to “intoxication” as discussed by another female backyard producer in Yaruqui, who said that when an animal is “intoxicated. … you can die with too much medicine,” so that traditional or “natural” alternatives are administered. Additionally, it was believed that antibiotic resistance leads to the use of stronger “chemical” products. Interestingly, few participants remembered where they had heard about antibiotic resistance, while some reported that this phenomenon had been discussed with neighbors or veterinarians.

Finally, the backyard producers mentioned that one of the most important consequences of overusing antibiotics is that they have observed that when a product is no longer effective, stronger alternatives have to be used:

[An animal] is already resistant to the medication you give him. For example, once you get sick with the flu and you use the same medicine and another time the same and the same … the same as a person (female producer, Yaruquí).

Veterinarian FGD participants reported that antibiotic resistance is a serious problem in Ecuador and that they have begun to witness cases in which infections in food animals are resistant to commonly used antibiotics, which results in the use of increasingly stronger alternatives that can be harmful to human health. They stated that the development of antibiotic resistance is due mainly to the indiscriminate use of antibiotics in animals for human consumption since they are often used for growth promotion and disease prevention in commercially oriented food animal production. They further stated that the problem goes hand in hand with a lack of awareness among veterinary shop owners, who are also responsible for the increase in antibiotic resistance because they are willing to sell antibiotics with little control and often without a prescription. Additionally, failure to use best practices (i.e., recommended doses and timing) may lead to overuse of antibiotics and subsequent resistance. As one veterinarian FGD participant explained:

Among large-scale producers where [veterinarians] have gone to give advice, it has been seen that [producers] do not use the full dosage. And when we do the calculations [we find that] they do not use even half the dose, and many times they want their birds to be healthy in two or three days. So, they continually change antibiotics. They are on sulfa and they change to quinolones and that’s how they change, until there are three or four antibiotics for the same disease, and logically it is because they do not dose well and do not leave enough time for the birds to be cured (male veterinarian, Quito, group 2).

The consequence of the misuse and overuse of antibiotics is the development of antibiotic resistance discussed above, such that the application of these products is increasingly ineffective and the effects on human populations and the environment become more problematic.

### Government policy regarding antibiotic use in agricultural production

Participants in the veterinarian FGDs confirmed the indiscriminate use of antibiotics among commercially oriented medium- and large-scale food animal producers for disease prevention and control and to promote growth in food animals. They attributed this growing problem to a lack of information by producers and even veterinarians as well as ineffective government control. One veterinarian explained his belief that the consequences have already been observed, but alternatives are not yet readily apparent:

I believe that there should definitely be more control, but many technicians and producers lack awareness of the impact of [antibiotic resistance]. Perhaps one of the disadvantages of indiscriminate or irresponsible use of antibiotics is that we run out of tools or strategies that could work in other conditions using an antibiotic responsibly, because I think that eliminating the use of an antibiotic by itself does not make sense because then how can we treat a disease that absolutely needs an antibiotic? (Male veterinarian, Quito, group 2).

Another veterinarian commented that government agencies are part of the problem; he believed that:

No, I think that it is necessary to point out that the indiscriminate use of antibiotics that has been taking place and is being regulated. It is generated from the state agency that now gives away kits of antibiotics and medications, where antibiotics, antiparasitics, hormones … are gifts to the producer without absolutely any control and without any explanation of how or when they should be used. (Female veterinarian, Quito, group 2).

The key informant from the Ministry of Agriculture and Livestock had a contrasting viewpoint, reporting on a government plan to address the problem of antibiotic resistance by regulating sales and monitoring, while admitting that the latter is a piecemeal effort because of limited resources. She also confirmed that the sale of Colistin, an antibiotic that had been widely used for growth, had been banned since the beginning of 2020. Finally, she believed that there is increasing recognition in government circles that antibiotic resistance is an important problem in Ecuador and must be systematically addressed:

As for whether we are aware of the importance of antibiotic resistance at the national level, that is why as a country we are obliged and had promised that in 2017 we were going to implement a national plan to mitigate antibiotic resistance. However, this plan, being national, had to link several ministries, the Ministry of Health, the Ministry of Agriculture, the Secretariat of Science and Technology, the Ministry of the Environment, and other entities. The signing was delayed but in August (2019), we had a national plan that involves (those) ministries (female key informant, Ministry of Agriculture and Livestock, Quito).

## Discussion

Small-scale food animal production constitutes a large and heterogeneous sector in Ecuador. Many of those in the coastal region implement commercially oriented production strategies, including the use of veterinary products that have been linked to antibiotic resistance [41]. In contrast, studies conducted in the study area have found that 20 percent of small-scale food animal producers use antibiotics [27, 28]. This paper analyzes the reasons for which the other 80 percent do not and finds that the explanation lies in their motivations and production strategies. The paper then links these strategies with public health issues related to antibiotic resistance.

The findings presented here suggest that these backyard food animal producers generally eschew the use of purchased inputs, especially antibiotics. They focus instead on keeping small numbers of food animals and poultry for non-commercial household consumption; lacking the economic motivation to increase productivity through the use of antibiotics, they prefer to use traditional veterinary remedies at little or no cost, while accepting fairly predictable amounts of loss.

Beyond these economic motivations, a second reason for which backyard producers avoid using antibiotics for disease prevention and control or for growth prevention is related to a sense of planetary health incorporated in traditional Andean cosmology. Historically, peasant farmers, indigenous households, and other small-scale producers in the Ecuadorian highlands and other parts of the Andean region have maintained a traditional view of the physical and spiritual environment that surrounds them, according to which, Mother Earth (*Pachamama*, in the Kichwa language) represents an all-encompassing state of balanced well-being. In this view, the earth is to be revered and protected as the foundation for individual, community, and planetary health [42, 43]. In this context, the use of antibiotics is viewed as harmful to human and animal health. This concept is enshrined as rights of nature in the Ecuadorian constitution [44]. This view is not unique; for example, unlike their conventional counterparts, organic food animal producers in the United States eschew the use of antibiotics as a matter of conscience as well as practicality [45]. The significance is that antibiotic resistance represents a growing threat to human and environmental health in Ecuador as it does throughout the world. The close proximity of food animals to workers and surrounding communities means that the spread of antibiotic resistant bacteria and potential associated threats do not require direct contact; children are particularly vulnerable to bacterial infection [46]. The threat is likely to grow as commercially oriented, large-scale industrial food animal production proliferates in Ecuador and throughout world. The problem is exacerbated by the practices of sales agents of veterinary products, who are interested principally in improving profits [27].

The threat of antibiotic resistance has not gone unnoticed; calls to action have drawn attention to the problem, and a variety of alternatives have been proposed [47–50]. This paper suggests that while antibiotic resistance has been associated with small-scale food animal production in some cases, alternative paths have also been followed. As they do in many parts of the world, limited resource backyard producers in highland Ecuador use a variety of traditional non-chemical remedies that are readily at hand in the household for the reasons discussed above [51]. Moreover, these rural households are reasserting the validity of their economic and cultural practices, which can be parlayed into the implementation of alternatives that promote human and environmental health. Thus, researchers, veterinary professionals, and decision makers would be well advised to understand the perceptions, attitudes, and practices of backyard producers with regard to the use and potential misuse of antibiotics in order to construct more sustainable alternatives [52, 53].

The findings presented in this paper are based on research that faced several limitations. This study was based on theoretical sampling and systematic data analysis [33–36] that allow for the identification of key dimensions of backyard food animal production. The findings nevertheless provide an alternative view of this phenomenon and invite further qualitative and quantitative research that could provide greater understanding of the perceptions, attitudes, and practices of this large but poorly understood group of rural inhabitants.

## Conclusions

The findings presented in this paper suggest an alternative to the dependence on veterinary antibiotics and to the growing public health threat of antibiotic resistance. The development and implementation of relevant policies and programs on a larger scale depend on understanding the motivations that guide backyard food animal producer strategies. Study participants described two reasons for which they do not depend on the use of veterinary antibiotics. First, their calculation of the relative costs and benefits of using antibiotics for disease control and prevention and for growth promotion differs from that of commercially oriented producers, who focus on maximizing profit and productivity. For backyard producers, keeping food animals is not a full-time income-generating activity; rather it is usually a part-time activity that contributes to household consumption.

Second, these backyard food animal producers retain a traditional reverence for interwoven threads of environmental and human health found throughout the Andean region. Centered on *Pachamama* or Mother Earth, Andean cosmovision is based on notions of balance, interconnectedness, health, and diet**.** Antibiotics, in this view, not only represent an unnecessary expense, but a threat to household, community, and environmental health.

Thus, these rural families, who live in the virtual shadow of Ecuador’s capital city, avail themselves of household food animal production, while participating in urban labor and commercial markets. Since they rarely use antibiotics, veterinarians have only a general notion of backyard food animal production strategies. As discussed above, these non-commercial producers rarely (if ever) solicit veterinarian services because they have their own methods of treating their animals and poultry and furthermore do not have resources to either pay for professional services or purchase medications that might be prescribed.

These findings suggest that the agendas of research, technical assistance, and decision-making should incorporate alternatives to antibiotic use and misuse. In this regard, the results presented in this paper suggest that it is essential to understand the heterogeneity of the small-scale food animal production sector and that in particular, backyard producers operate with a logic that lies beyond profit and productivity and incorporates alternatives to the use of antibiotics.

## Data Availability

The datasets generated during and analyzed during the current study are not publicly available in order to protect the anonymity of the participants but are available from the corresponding author on reasonable request.
